# Repeated exposure to novelty promotes resilience against the amyloid-beta effect through dopaminergic stimulation

**DOI:** 10.1007/s00213-024-06650-5

**Published:** 2024-08-15

**Authors:** Cintia Velázquez-Delgado, Eduardo Hernández-Ortiz, Lucia Landa-Navarro, Miguel Tapia-Rodríguez, Perla Moreno-Castilla, Federico Bermúdez-Rattoni

**Affiliations:** 1 División de Neurociencias, Instituto de Fisiología Celular, Universidad Nacional Autónoma de México, 04510 Mexico City, Mexico; 2 Instituto de Investigaciones Biomédicas, Universidad Nacional Autónoma de México, 04510 Mexico City, Mexico; 3 Laboratory of Cognitive Resilience, Center of Aging Research (CIE), Center for Research and Advanced Studies of the National Polytechnic Institute, CINVESTAV, Mexico City, Mexico

**Keywords:** Alzheimer’s disease, Beta-amyloid peptide, Hippocampus, Catecholamines, Cognitive reserve, Resilience, Spatial memory

## Abstract

**Rationale:**

The accumulation of beta-amyloid peptide (Aβ) in the forebrain leads to cognitive dysfunction and neurodegeneration in Alzheimer's disease. Studies have shown that individuals with a consistently cognitively active lifestyle are less vulnerable to Aβ toxicity. Recent research has demonstrated that intrahippocampal Aβ can impact catecholaminergic release and spatial memory. Interestingly, exposure to novelty stimuli has been found to stimulate the release of catecholamines in the hippocampus. However, it remains uncertain whether repeated enhancing catecholamine activity can effectively alleviate cognitive impairment in individuals with Alzheimer's disease.

**Objectives:**

Our primary aim was to investigate whether repeated exposure to novelty could enable cognitive resilience against Aβ. This protection could be achieved by modulating catecholaminergic activity within the hippocampus.

**Methods:**

To investigate this hypothesis, we subjected mice to three different conditions—standard housing (SH), repeated novelty (Nov), or daily social interaction (Soc) for one month. We then infused saline solution (SS) or Aβ (Aβ_1-42_) oligomers intrahippocampally and measured spatial memory retrieval in a Morris Water Maze (MWM). Stereological analysis and extracellular baseline dopamine levels using in vivo microdialysis were assessed in independent groups of mice.

**Results:**

The mice that received Aβ_1-42_ intrahippocampal infusions and remained in SH or Soc conditions showed impaired spatial memory retrieval. In contrast, animals subjected to the Nov protocol demonstrated remarkable resilience, showing strong spatial memory expression even after Aβ_1-42_ intrahippocampal infusion. The stereological analysis indicated that the Aβ_1-42_ infusion reduced the tyrosine hydroxylase axonal length in SH or Soc mice compared to the Nov group. Accordingly, the hippocampal extracellular dopamine levels increased significantly in the Nov groups.

**Conclusions:**

These compelling results demonstrate the potential for repeated novelty exposure to strengthen the dopaminergic system and mitigate the toxic effects of Aβ_1-42_. They also highlight new and promising therapeutic avenues for treating and preventing AD, especially in its early stages.

**Supplementary Information:**

The online version contains supplementary material available at 10.1007/s00213-024-06650-5.

## Introduction

Alzheimer's disease (AD) is a neurodegenerative disorder characterized by progressive cognitive impairment, such as memory loss and spatial disorientation (Selkoe 2000). Clinical observations have led to the identification of two forms of AD. Early-onset AD, observed among individuals younger than 60 years old, accounts for 6% of all cases and is linked to genetic factors (Zhu et al. 2015). Conversely, the second form is sporadic, comprising most AD cases. In this regard, two pathophysiological markers have been related to AD: the accumulation of amyloid-beta peptide (Aβ) in oligomers and plaques and hyperphosphorylated tau neurofibrillary tangles (Boncristiano et al. 2005). Although clinical and pre-clinical evidence has also been found that the Tau protein and Aβ plaques may be linked to AD cognitive symptoms (Roda et al. 2022), there is no direct link between the Aβ plaque burden and cognitive performance. In this context, several works have found that the amyloid plaques are not the main toxic form (Gouras et al. 2010; Mucke and Selkoe 2012; Pozueta et al. 2013) and the Aβ deposition does not impair cognitive performance (Boncristiano et al. 2005; Pike et al. 2007). Interestingly, animal models of AD that only produce oligomeric forms of Aβ exhibit cognitive impairment that precedes Tau protein hyperphosphorylation (Tomiyama et al. 2010; Lasagna-Reeves et al. 2010). Therefore, it has been proposed that the oligomers of Aβ, instead of the plaque deposition, promote the toxic effects underlying cognitive impairments in the early stages of AD (Benilova et al. 2012).

Late-onset AD clinical and histopathological analyses have shown that environmental factors may delay AD emergence and development. Recently, evidence showed that some cognitively normal elderly individuals harbor Aβ in their brains, with a similar accumulation degree as patients with a post-mortem AD diagnosis (Roe et al. 2007). Therefore, the cognitive reserve is one of the mechanisms that create resilience and is defined by the brain's ability to maintain cognitive performance even in the presence of brain injury or aging (Stern 2006; Stern et al. 2023). Cognitive reserve points out that experiences across the lifespan can overcome a pathological burden to decrease cognitive function. Lifetime parameters such as education level, social interaction, and cognitive challenges, may be related to inducing brain adaptations that could represent a neuronal substrate of cognitive reserve. However, assessing the effects of these lifetime parameters on the brain is challenging, and appropriate controls and longitudinal measures should be considered for comparison purposes. In this regard, animal environmental enrichments have been employed to study cognitive benefits in people affected by neurodegenerative diseases such as AD (Hannan 2014; Petrosini et al. 2009).

Previous studies in AD animal models have found that cognitive stimulation through environmental enrichment is related to decreased memory impairments in an AD mouse model (Costa et al. 2007; Jankowsky et al. 2005; Arendash et al. 2004). Moreover, these protocols encompass several factors that should be identified to evaluate their benefits, such as the level of novelty and environmental complexity (Nithianantharajah and Hannan 2006). Particularly, evidence has emphasized that repeated exposure to novel stimuli is crucial for improving cognitive function in an AD mouse model (Veyrac et al. 2009). In this context, it has been demonstrated that exposure to novelty may mitigate the toxic effects of Aβ oligomers in vitro by triggering the expression of normal long-term plasticity mediated by the noradrenergic system (Li et al. 2013). Additionally, some studies have found that the catecholaminergic system in the hippocampus supports this enhanced plasticity induction (Li et al. 2003; Moncada and Viola 2007), and the detection of contextual novelty depends on the release of dopamine (DA) and noradrenaline (NA) in the CA1 hippocampus (Moreno-Castilla et al. 2017; Moreno-Castilla et al. 2016; Lisman and Otmakhova 2001; Gálvez-Márquez et al. 2022; Bastin et al. 2019; Chen et al. 2023). Altogether, this evidence suggests that the novelty may be a factor that possibly triggers enhanced catecholaminergic activity, preventing deficits in synaptic plasticity and contextual memory from the toxic effects of Aβ.

Numerous studies have implicated dysfunction in the catecholaminergic system in the development of AD (Martorana and Koch 2014; Guzmán-Ramos et al. 2012; Pillet et al. 2020). In this context, significant DA and NA level reductions have been observed in cortical and subcortical brain regions in both human and AD mouse models. These reductions correlate with decreased TH + markers and memory impairment (Moreno-Castilla et al. 2016; Pillet et al. 2020; Nobili et al. 2017; La Barbera et al. 2021; Himeno et al. 2011; Gutiérrez et al. 2022). In the AD mouse models, reduced hippocampal innervation from the midbrain's dopaminergic neurons is related to neuronal excitability modifications and aberrant gamma-wave activity (Spoleti et al. 2024) leading to impairments in episodic memory formation (Griffiths and Jensen 2023). Similarly, noradrenergic neurons in the locus coeruleus are progressively impaired in AD humans (Kelly et al. 2017) and transgenic rodent models (Flores-Aguilar et al. 2022; Oikawa et al. 2010; Sakakibara et al. 2021). Treating with NA precursor or DA agonists has been shown to reduce the toxic effects of Aβ and improve performance in spatial memory tasks (Himeno et al. 2011; Kalinin et al. 2012). Additional experiments have revealed that blocking DA reuptake in the insular cortex of AD transgenic mice can mitigate memory recognition impairment, and increase dopaminergic activity necessary to convert cortical long-term depression induced by Aβ_1-42_ oligomers to long-term potentiation (Moreno-Castilla et al. 2016; Guzmán-Ramos et al. 2012). Together, these studies highlight that catecholaminergic activity is closely related to AD dysfunction, representing a putative neurochemical substrate of cognitive reserve against the effects of Aβ.

**Fig. 1:**
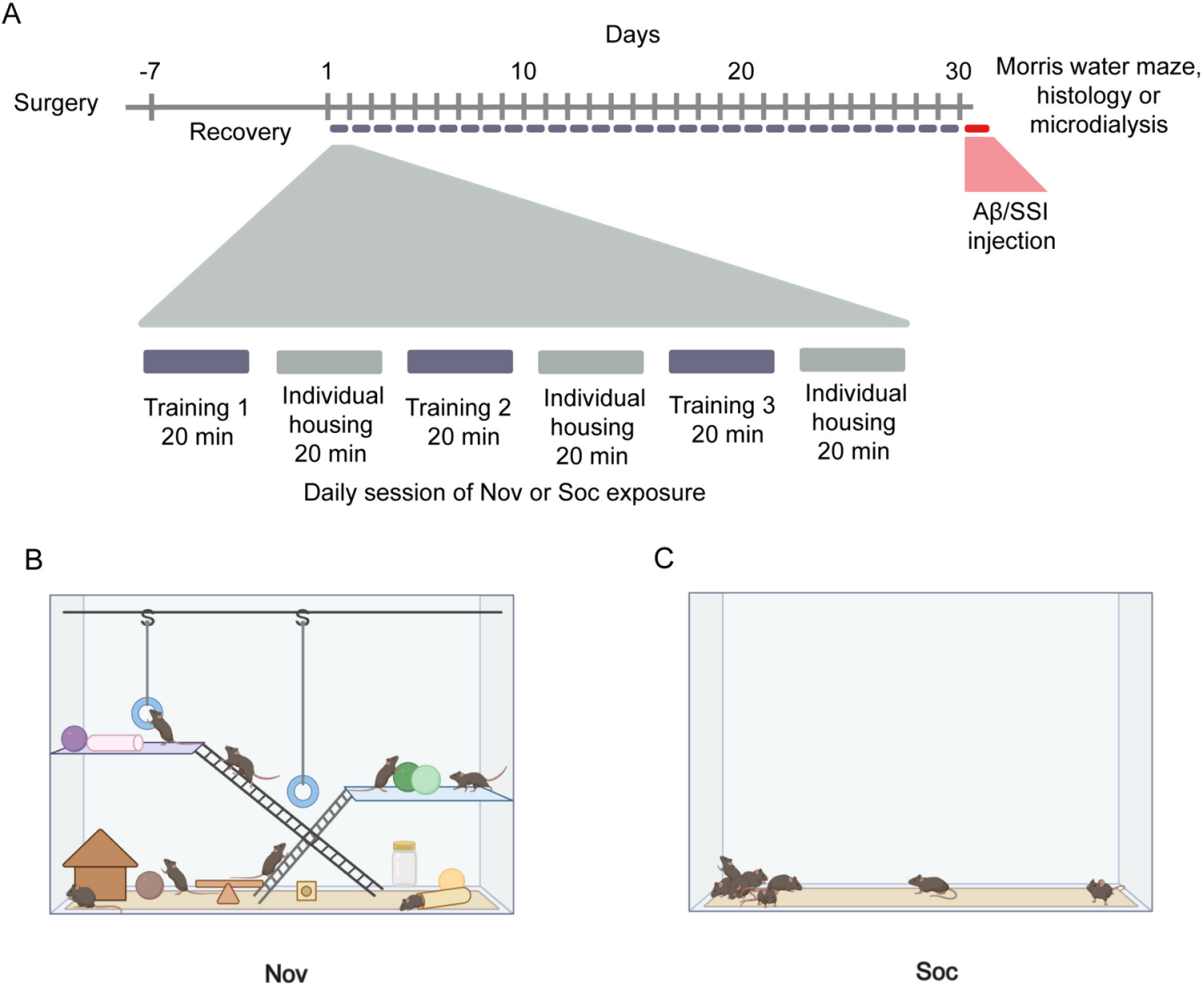
Shows the behavioral protocol employed. A) Depict the timeline of the behavioral protocol. B) Representative scheme of the Nov training maze employed. C) Scheme of Soc condition maze without novelty stimuli

In the present study, we hypothesized that repeated exposure to novelty stimuli enhances catecholaminergic activity, protecting against the toxic effects caused by the administration of Aβ_1-42_ oligomers in the hippocampus CA1. To assess this hypothesis, we measured the impact of an acute administration of Aβ oligomers in the hippocampus on a spatial memory task after standard housing (SH), social interaction (Soc), and repeated novelty (Nov) conditions. Next, through stereological quantification, we measured the length of catecholaminergic fibers after this protocol. Finally, employing in vivo microdialysis, we measured the basal levels of catecholamines within the hippocampus after these conditions (Fig. 1). Our results demonstrate that an enhanced dopaminergic system after constant novelty exposure may prevent the toxic effects of Aβ_1-42_ oligomers, supporting the cognitive reserve hypothesis.

## Methods and materials

### Animals

This study used B6129SF2/J WT male mice aged between 4 and 5 months. The mice were individually housed at 22–24 °C with a 12/12 h light/dark cycle. Water and food were supplied ad libitum. All experiments were performed by the current National Norm for the Use of Animals (NOM-062-ZOO-1999) and with the approval of the Care and Use of Laboratory Animals of Instituto de Fisiología Celular, UNAM (FBR30-14).

### Surgical procedures

Mice were anesthetized with 4% isoflurane and fixed on a stereotaxic apparatus using a mouse adaptor and they were maintained under anesthesia ~ 0.5–1.0%. For microinjections of Aβ into the hippocampus, we implanted two 23-gauge stainless steel cannula over the dorsal hippocampus (DV -1.0 mm; AP = -2.35; ML =  ± 0.5 mm; 9 mm long; Small Parts, Logansport, IN, 81 USA; Figure S2) and they were secured with small screws and dental acrylic. A dummy cannula (33-gauge, 12 mm) was inserted into the guide cannula to prevent clogging. Antibiotics (polymyxin B and iodine) were dosed locally. For microdialysis experiments, we implanted a guide cannula over the CA1 dorsal hippocampus in a counterbalanced manner (DV -1.2 mm; AP = -3.0; ML =  ± 2.0, 20° angle; CMA/7 Microdialysis (Solna, 80 Sweden); histological analyses to locate cannula placement were performed after the mice were euthanized (see below).

### Preparation of Aβ oligomers

Aβ_1-42_ oligomers (Aβ) (Millipore; Temecula, CA) were treated with NaOH. The powder was diluted in NH_4_OH to obtain a final 1 mg/mL concentration. This solution was sonicated for one minute to promote the oligomeric formation (Moreno-Castilla et al. 2016; Fezoui et al. 2000). Then, the Aβ oligomers were prepared by diluting the stock preparation into the saline solution and incubating for 4 days at 37 °C. This protocol was also applied for a scrambled Aβ sequence (Scr, Bubendorf, Switzerland) used as a control peptide for Aβ (Moreno-Castilla et al. 2016). A 0.5 μL volume of either 7.74 μM Aβ or Scr solutions was bilaterally dosed at the CA1 region of the hippocampus at a 0.25 µL/min rate using a 30-ga needle protruding 500 µm from the tip of the guide cannula.

### Behavioral protocols, spatial memory tasks, and Aβ injections

We designed a Nov protocol that repeatedly exposed mice to different objects and contexts. They were allocated in groups of 4–6 mice and placed inside a Kaytee CritterTrail Habitat® (Costa et al. 2007). Several toys with different shapes were placed inside the Kaytee CritterTrail Habitat®, and the animals were allowed to explore it for 20 min. After this period, all mice were placed back into their respective vivarium cage for another 20 min, also called a Nov or Soc exposure cycle. This step was repeated two more times and the objects were changed between every cycle (Fig. 1). The total Nov protocol lasted 30 days. The animals remained together in the same arena task during the Nov protocol. Thus, we decided to isolate this factor and evaluated whether the groups formed by animals may impact the results. Based on this factor, we established a group with Soc conditions. These conditions consisted of mice placed in a box in groups of 4–6, and social interaction was allowed during fixed and identical periods as the Nov protocol. All procedures were followed similarly but without exposing them to novel objects. We used control SH conditions for all experiments in which animals were kept in their home cage throughout the experiments (Fig. 1).

**Fig. 2:**
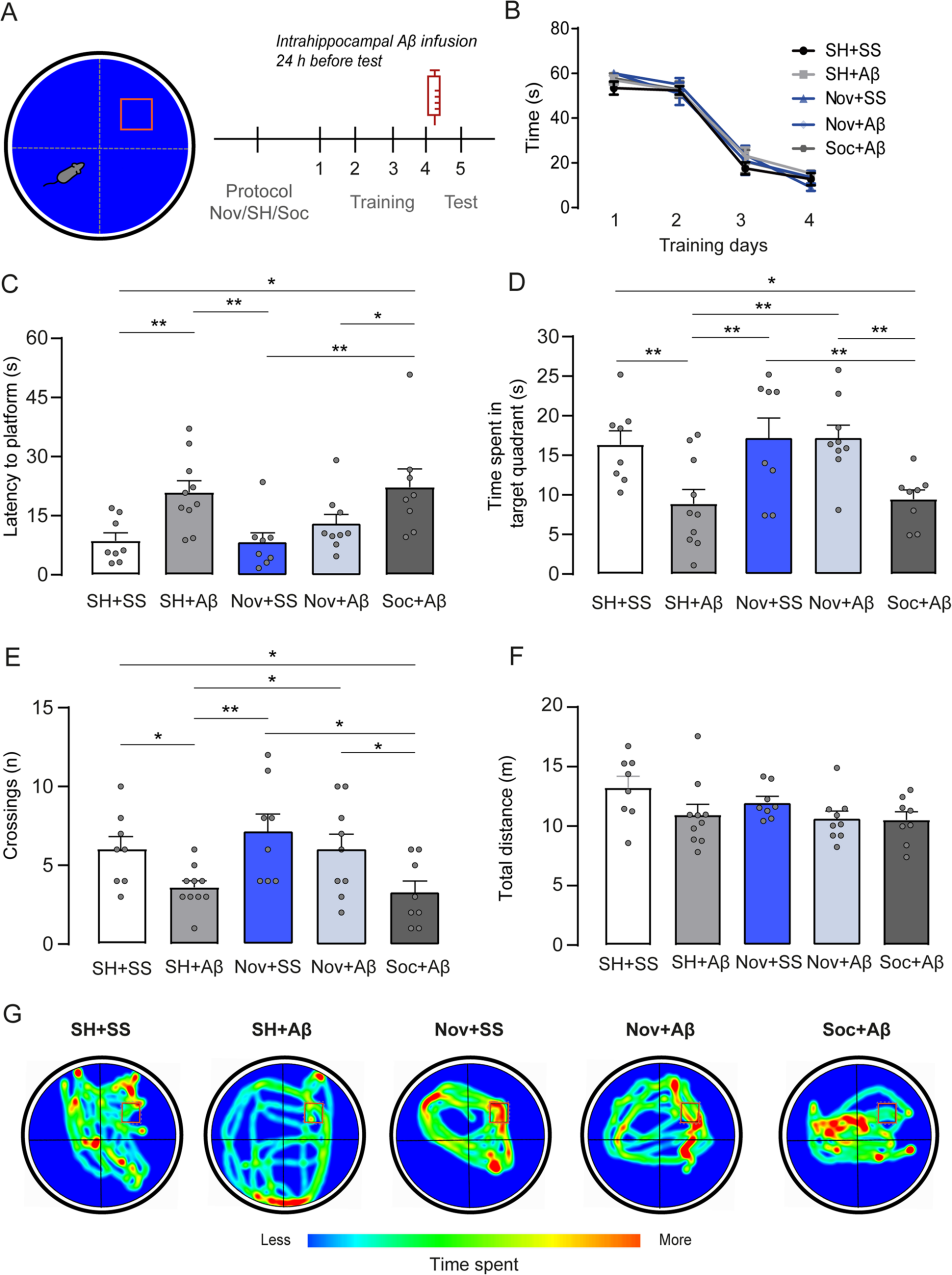
The acute administration of Aβ impairs spatial memory but repeated novelty exposure maintains memory performance. **a** Schematic representation of the Spatial MWM and administration of saline solution or Aβ_1-42_ 24 h before the test in SH + SS and SS + Aβ; Nov + SS and Nov + Aβ; and Soc + Aβ, groups. **b** Latency to the platform during training. **c** Latency to the platform during test day. **d** Time spent in the target quadrant. **e** Number of crossings to the place of platform location during test day. **f** Distance was recorded for all groups during the memory test. **g** Representative heat maps of a mean trajectory of the mice in each group; the blue color represents less time spent on exploration, and the red color shows more time spent on exploration. All data are shown in ± SEM, *p < 0.05, **p < 0.01

**Fig. 3:**
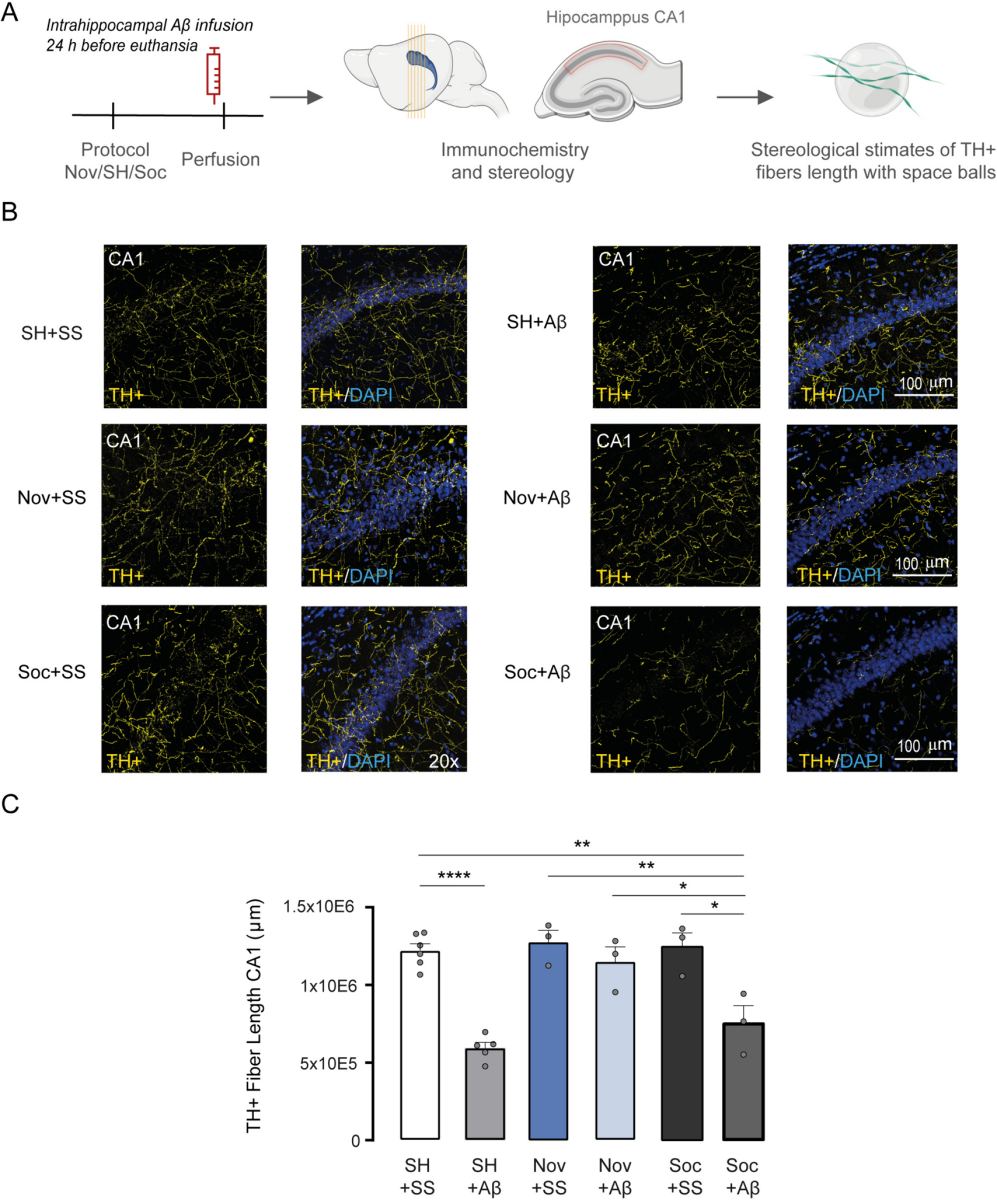
The acute administration of Aβ reduces the length of the TH + fibers, and novelty prevents its reduction. **a** Depict the timeline of the behavioral protocol before the saline solution or Aβ infusion for SH + SS or SS + Aβ; Nov + SS or Nov + Aβ; and Soc + SS or Soc + Aβ. Subsequent procedure to immunohistology and stereological analysis. **b** Representative micrographs of the immunohistochemical to TH + for administration of saline solution or Aβ_1-42_ 24 h before the sacrifice. **c** Total length of the TH + fibers in the dorsal hippocampus. All data are shown in ± SEM, *p < 0.05, **p < 0.01, ***p < 0.001, ***p < 0.0001

After the animals were exposed to the Nov, Soc, or SH training, they were measured for spatial memory performance. All animals were trained in a Morris Water maze (MWM) to evaluate spatial memory using a 1.10 m diameter circular tank filled with white non-toxic water at 20-22ºC (Fig. 2). During the acquisition days, the mice were trained to locate a hidden platform employing two opposite distal cues signaled in the wall of the MWM. On the first training day, each mouse was placed in a different quadrant section of the tank and randomly swam until the platform was found. They learned the location of a hidden platform by using intra- and extra-maze cues. If a mouse failed to find the platform within 6 s, the experimenter helped by placing it on the platform for 20 s. This procedure was repeated four times for each animal for four days. Each animal takes a time out between assays for 1 min. Once the behavioral protocols were completed, the Nov and Soc groups were divided in two. Half of the animals of each group received 0.5 μL of Aβ solution, and the other half 0.5 μL of saline solution (SS), was directly injected into the hippocampus 24 h before conducting the test session of the Long-Term Memory (LTM) test. All groups were evaluated for their performance in the MWM retrieval, consisting of 60 s to find the platform’s location. The spatial memory test was recorded with a camera and analyzed using the ANY-maze tracking software. To explore the effect of Nov against the Aβ oligomers in the length of the TH + terminals, we used an independent cohort of animals: SH + SS as a control group, SH + Aβ; Nov + SS; Nov + Aβ; Soc + SS; and Soc + Aβ, as described but without spatial training to measure the length of the TH + terminals in CA1. Finally, other cohorts of animals were used to calculate the basal neurotransmitter baselines in the hippocampus-CA1 after following the correspondent SH + SS, SH + Aβ, Nov + SS, and Nov + Aβ protocols and 24 h later, were submitted to in vivo microdialysis protocol (Fig. 3).

**Fig. 4:**
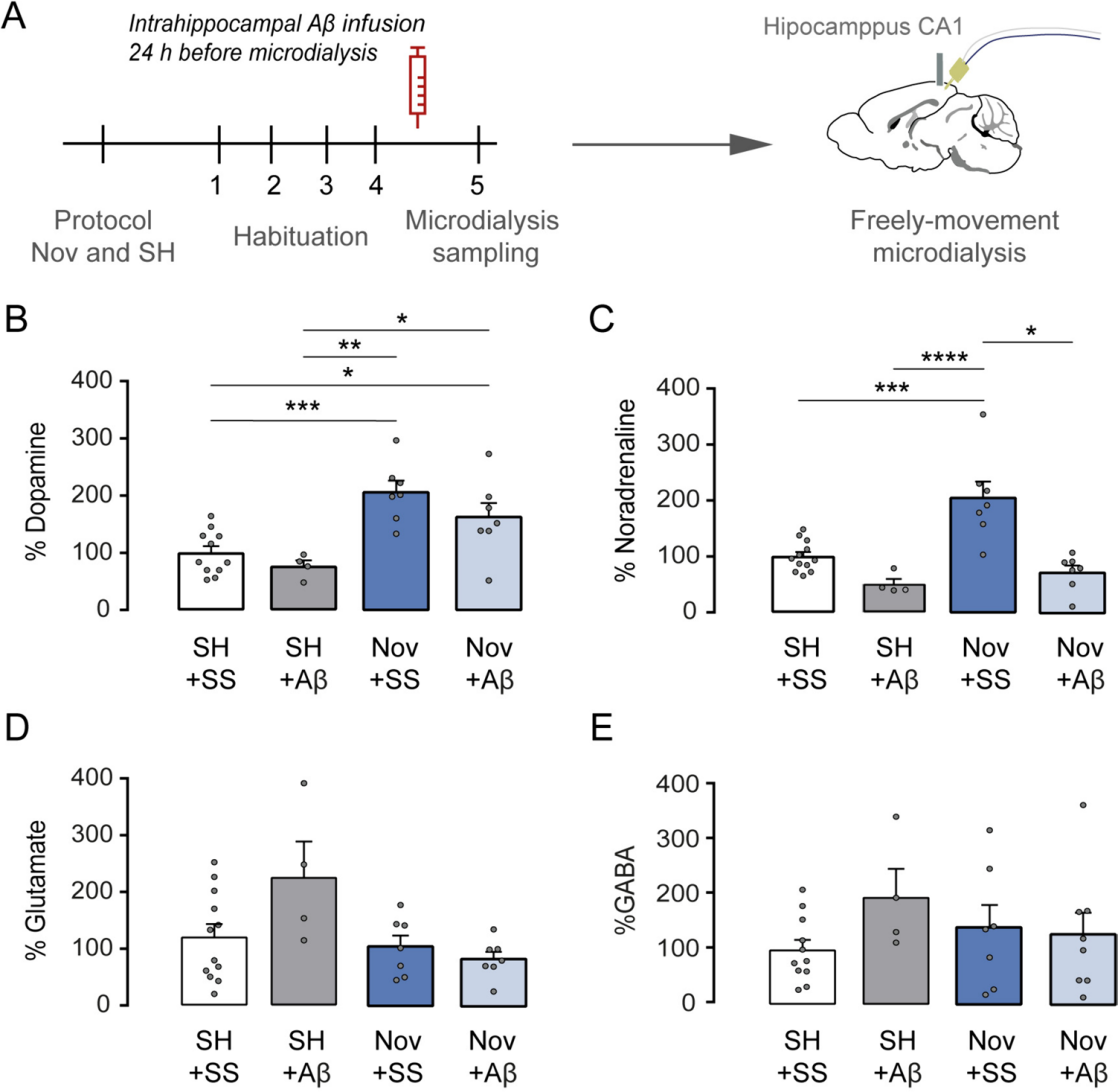
Novelty exposure improves the baseline of dopamine and noradrenaline, preventing the toxic effects of intrahippocampal Aβ administration. **a** It depicts the timeline of training protocol and the in vivo microdialysis procedure for SS + SS or SS + Aβ; Nov + SS or Nov + Aβ groups. **b** Shown the basal DA extracellular levels percentage, **c** NA, D) Glu, and E) GABA within the hippocampus CA1 after training protocols and saline or Aβ_1-42_ infusion. All data are shown in ± SEM. *p < 0.05, **p < 0.01, ***p < 0.001, ****p < 0.0001

Although the behavioral results were presented first for explanatory convenience, the experiments were conducted following a specific sequence. We began by using stereological quantifications to measure the length of TH + after infusing Aβ_1-42_ oligomers or SS after SH, Nov, and Soc conditions. Upon analyzing the stereological findings, we found no significant effect of Soc + Aβ on the length of TH + fibers compared to the control Soc + SS or SH + SS groups (Fig. 3C). Consequently, we decided not to evaluate the Soc + SS group in spatial memory performance (Fig. 2B). Similarly, based on our MWM experiments, we did not observe any improvement in spatial memory for the Soc + Aβ group. Hence, we only assessed the extracellular concentrations of neurotransmitters in the SH + SS, SH + Aβ, Nov + SS, and Nov + Aβ groups (Fig. 4A). These experimental designs were planned to minimize the use of experimental animals and to uphold ethical considerations in preclinical assays (Kiani et al. 2022).

### Microdialysis and capillary electrophoresis protocols

Microdialysis membranes (CMA/7 Microdialysis, Solna, Sweden) of 2 mm were inserted in the 110-guide cannula placed into the dorsal hippocampus. An automatic micro-infusion pump perfused the ringer solution (118 mM NaCl, 4.7 mM KCl, and 2.5 mM CaCl2) at 0.25 μL/min. We perfused the dorsal hippocampus through a cuprophane semipermeable microdialysis membrane (6 Kda pore). We employed syringes of 1 mL over an injection pump to infuse the ringer solution (100 pump CMA Microdialysis; Kista, Sweden). After stabilizing for 60 min, seven samples of 4 μL were collected every 16 min at 0.25 μL/min. All the samples were taken to assess the baseline levels and stored at -80 °C until further analysis was required. The samples were stored with 1 μL of antioxidant solution (25 mL L-ascorbic acid, 27 mM Na2EDTA, and 1 M acetic acid). All samples were derivatized with 5-furoylquinoline-3-carbaldehyde (FQ, 16.6 mM, Molecular Probes; Invitrogen, USA) and were analyzed by capillary electrophoresis coupled to a laser-induced fluorescence detector (Beckman-Coulter PACE/MDQ, Glycoprotein System CA, USA.) as previously described (Hernández-Ortiz et al. 2023). The catalysis was made through 2 μL KCN (24.5 mM) in borate buffer (10 mM, pH 9.2). We added 1 μL of an internal standard (0.075 mM, O-methyl-L-threonine; FULKA, USA). This solution was heated at 65 °C for 15 min without light. The detection of the fluorophore was using a laser light coupled (λ = 488 nm, argon-ion) to a capillary electrophoresis system and represented in an electropherogram and further analyzed in 32 Karat TM 8.0 software (P/ACE MDQ, Beckman Coutler; Pasadena, USA). The samples were detected by migration period at 25 kV, and hydro-dynamically injected into a capillary system using 0.5 psi for 5 s in a borate buffer (borates 35 mM, sodium dodecyl sulfate 25 mM, and 13% methanol HPLC level, pH 9.6).

### Histological and immunohistochemical analyses

Animals were euthanized with a pentobarbital (PiSA, Mexico) lethal dose, and they were transcardially perfused with 0.9% NaCl followed by 4% paraformaldehyde, pH 7.4. The brains were removed and post-fixed for 24 h to be later transferred to 30% sucrose. The whole brain was embedded in a tissue-tek medium and coronally sectioned using a cryostat with 35 µm thickness (Leica et al., USA). Free-floating sections were processed as described previously in Moreno-Castilla et al. 2016 (Moreno-Castilla et al. 2017). Overnight incubation was performed with a primary rabbit polyclonal antibody raised against TH (1:1000; Pel-Freez, Rogers, AR) at 4ºC. Then, another incubation was performed with a secondary conjugated CY3-goat anti-rabbit IgG (1:250; Millipore, Darmstadt, Germany) for 90 min at RT. The subsequent sections were washed and mounted on Super-frost Plus micro slides (VWR, Leuven, Belgium) to perform a stereological quantification of the TH-immunoreactive fiber length.

### Stereological quantification

The area of interest was limited within the CA1 area of the hippocampus at the anterior–posterior axis (from bregma: AP -1.45 to -3.58 and DV = -0.8 to -1.2 mm range) (Atlas and : Allen Brain Atlas: Mouse Brainhttps:, , mouse.brain-map.org, static, atlas. xxxx). We analyzed regions of interest from the subiculum CA1 to the dorsal hippocampus CA1. The region of interest was traced at a 4X magnification based on the Allen Mouse Brain Atlas; for each section, unbiased stereological length estimates and space ball procedure was performed. To measure the length of the TH positive axonal process within the hippocampus, we combined the optical fractionator with a space balls probe focused on the axon, dendrite, and capillary length (West 2018). We employed this probe to avoid the inherent anisotropy in biological tissues at the quantification sites (West 2018). We stimated the nerve fiber length across the CA1 layer in serial sections of mice’s hippocampus for both control and experimental groups. Only those TH + fibers showing defined and continuous staining across the Z-axis were considered in our counting. Space balls sampling was conducted out using a 25 μm-radius hemisphere probe within a 22,500 μm2 grid area and a section periodicity value 5. Counting was done by using an Olympus BX51WI microscope (Olympus Corporation, Tokyo, Japan) equipped with a Disk Scanning Unit (Olympus), an XYZ motorized stage (Ludl Electronics Products, Hawthorne, NY, USA) and a Hamamatsu EM-CCD camera (C9100-02, Hamamatsu Photonics K.K., Shizuoka, Japan); segments were evaluated with a UPlanSAPO 60X N.A.1.20 water immersion objective (Olympus). The assembly was controlled by the Stereo-Investigator software (v.9.0.1, MBF Bioscience, Williston, VT, USA) located at Unidad de Microscopia IIBO-UNAM, RRID: SCR_022204. Gundersen’s error coefficient for stereological estimates was always below 0.10 for all count values.

### Statistical analysis

A multifactorial ANOVA was used to analyze the neurotransmitter levels. A one-way ANOVA was used to evaluate histological analysis and behavioral scores, followed by the post hoc tests. We used a significance value of p < 0.05. All data are shown in ± SEM. A two-way ANOVA was used to analyze the learning curve of WMM. We employed the -post hoc*-* Fisher test for all ANOVA analyses and- the *t-*student test in comparison when they were required.

## Results

Because exposure to Nov prevents deficits in spatial memory induced by Aβ_1-42_ oligomers, and previous reports have indicated that Nov increases dopaminergic activity (Moreno-Castilla et al. 2016; Nobili et al. 2017; Himeno et al. 2011; Kalinin et al. 2012), we tested the hypothesis that repeated Nov may prevent cognitive damage caused by Aβ. After exposing the animals to SH, Nov, and Soc conditions, we evaluated long-term spatial memory performance in a Morris Water Maze (MWM). After the last training trial, the animals were infused into the dorsal hippocampus with an SS or Aβ and divided into SH + SS, n = 8; SH + Aβ, n = 9; Nov + SS, n = 8; Nov + Aβ, n = 9; and Soc + Aβ, n = 8; groups (Fig. 2A, S2). On the acquisition days, all groups showed a gradual learning curve. The primary operational measure determining correct spatial learning in this paradigm is the lower latency to reach the platform across the four days (Curdt et al. 2022). As expected, our analysis showed no differences among groups throughout the days. We observed a shorter arrival time at the platform for all groups equally across the training (Fig. 2B; two-way repeated measures analysis of variance [ANOVA] did not show a group effect: F (4, 156) = 0.612, p = NS; but a time effect: F (3, 156) = 240.9, p < 0.0001; with no interaction: F (12, 156) = 0.503, p = NS; Fig. 2B). This data shows correct learning due to a progressive decrease in latencies across the days in the MWM task for all mice groups.

One day after receiving saline or Aβ, we evaluate the latency to reach the platform in all groups on the test day. Our analysis showed differences between all treated mice on the test day (one-way ANOVA showed significant differences among groups, F (4, 38) = 4.874, p < 0.01). The post hoc test reveals that the SH + SS control group showed less time to arrive at the platform than the SH + Aβ group (SH + SS vs. SH + Aβ, p < 0.01). Similarly, the Soc + Aβ group took more time to reach the platform location when compared with the SH + SS control group (SH + SS vs. Soc + Aβ, p < 0.05; Fig. 2C). In contrast, the Nov + SS mice took less time to arrive at the target position than SH conditions with Aβ1 (Nov + SS vs. SH + Aβ, p < 0.01, Fig. 2C) and Soc + Aβ (Nov + SS vs. Soc + Aβ, p < 0.01). Interestingly, the Nov Aβ group showed less latency time to reach the platform position than Soc + Aβ (Nov + Aβ vs. Soc + Aβ, p < 0.05, Fig. 2C). Notably, the Nov + SS group did not show significant differences compared to the SH + SS control mice (p = NS) and the Nov + Aβ group (p = NS; Fig. 2C).

We assessed the total time spent in the target quadrant containing the platform. Our results reveal significant differences across all groups on the test day (one-way ANOVA among groups, F (4, 38) = 5.279, p < 0.01). We observed that the SH + SS control group spent more time in the target quadrant than the SH animals treated with Aβ_1-42_ (The post hoc test showed differences to SH + SS vs. SH + Aβ, p < 0.01) and Soc + Aβ mice (SH + SS vs. Soc + Aβ, p < 0.05; Fig. 2D). Furthermore, we measured the time spent in the target quadrant for mice that received SS within the hippocampus and were previously exposed to the Nov protocol. Our analysis found that the group Nov + SS explored a similar time in the target quadrant as the SH + SS control condition (SH + SS vs. Nov + SS; p = NS, Fig. 2D). However, when comparing the time spent in the target quadrant, the Nov + SS showed more exploration time than the SH + Aβ mice (SH + Aβ vs. Nov + SS, p < 0.01, Fig. 2D) and the Soc + Aβ condition (Nov + SS vs. Soc + Aβ, p < 0.01, Fig. 2D). Moreover, the animals exposed to the Nov protocol previously treated with an intrahippocampal Aβ_1-42_ infusion showed more time within the target quadrant than the SH + Aβ group (SH + Aβ vs. Nov + Aβ, p < 0.01, Fig. 2D) and Soc + Aβ mice (Nov + Aβ vs. Soc + Aβ, p < 0.01, Fig. 2D). The latency data to arrive at the platform position and the time spent in the target quadrant corroborated that Nov, but not Soc conditions, is related to preventing impairments in spatial memory retrieval.

Additionally, we evaluated the number of crossings to the platform site on the test day and observed significant differences among groups (one-way ANOVA, F (4, 38) = 4.037, p < 0.01). The post hoc test showed a higher crossing number for animals in the SH + SS control condition than SH + Aβ (SH + SS vs. SH + Aβ, p < 0.05, Fig. 2E) and Soc + Aβ groups (SH + SS vs. Soc + Aβ, Fig. 2E). Similarly, we observed that the mice previously exposed to the Nov protocol showed more crossing numbers compared with the SH + Aβ group (SH + Aβ vs. Nov + SS, p < 0.01) and the Soc + Aβ infusion (Nov + SS vs. Soc + Aβ mice, p < 0.05, Fig. 2E). Notably, animals subjected to the Nov + Aβ protocol showed a similar crossing number as the SH + SS (SH + SS vs. Nov + Aβ, p = NS) and Nov groups (Nov + SS vs. Nov + Aβ, p = NS, Fig. 2E). Overall, this data suggests that Nov, but not Soc, prevents spatial memory recall impairment induced by Aβ.

Finally, we measured the total distance traveled by all groups of mice during the memory test. We did not observe changes in the total traveled distance among the groups (one-way ANOVA, F (4, 38) = 0.7627, p = NS, Fig. 2F), meaning that Aβ_1-42_ oligomers administration did not seem to produce motor alterations. In addition, we represented the memory test trajectories in heat maps, showing how Aβ_1-42_ oligomers can impair the retrieval of the exact location of the platform in the SH + Aβ and Soc + Aβ. In contrast, the Nov conditions groups, Nov + SS and Nov + Aβ showed better localization memory for the platform place (Fig. 2G). The overall findings indicate that the single administration of Aβ_1-42_ within the dorsal hippocampus had a significant impact on spatial memory retrieval, and the Nov environment, but not the Soc mice, was able to mitigate this cognitive impairment.

## Exposure to novelty prevents the reduction of TH + terminals after the administration of Aβ_1-42_ oligomers in the *hippocampus*

Several studies have found that DA neurons are susceptible to the toxic effects of Aβ_1-42_, and their axonal processes are diminished in Alzheimer’s disease animal models (Moreno-Castilla et al. 2016; Nobili et al. 2017). Therefore, we hypothesized that the repeated presentation of Nov could prevent decrements in TH + fibers. To evaluate whether TH + terminals would be preserved by Nov after Aβ_1-42_ administration, we performed immunohistochemical staining and unbiased quantifications to measure the TH + length fibers in the hippocampus CA1. To assess the TH + preservation, we formed an independent cohort of mice named SH + SS control group, n = 6; SH + Aβ, n = 5; Nov + SS, n = 3; Nov + Aβ, n = 3; Soc + SS, n = 3; and Soc + Aβ, n = 3 (Fig. 3A).

For this purpose, we employed stereological probes, an optical fractionator, and a dissector combined with the space balls probe. This approach is characterized by unbiased three-dimensional quantification which allows an estimate of the total length of axonal fibers (Larsen 2017; Gundersen 1986). We observed that Aβ_1-42_ oligomers diminished the length of TH + axons in the hippocampus CA1 in the SH + Aβ group compared with the SH + SS control group (one-way ANOVA, F (5, 17) = 19.470, p < 0.0001; post hoc test, SH + SS vs. SH + Aβ, p < 0.0001, Fig. 3B). Notably, the Nov + Aβ group was the only one that maintained the length of TH + axons compared with either the SH + Aβ (Nov + Aβ vs. SH + Aβ, p < 0.0001) or Soc + Aβ (Nov + Aβ vs. Soc + Aβ, p < 0.001). Interestingly, the Soc + Aβ group showed less TH + axonal length than SH + SS (SH + SS vs. Soc + Aβ, p < 0.001) and the Soc + SS group (Soc + SS vs. Soc + Aβ, p < 0.0139). However, our Nov protocol condition contributes to preventing the Aβ_1-42_ damage of the TH + fibers in the hippocampus CA1. It is important to note that the Nov condition did not increase the length of the TH + fibers above the SH conditions (Nov + SS vs. SH + SS, p = NS), suggesting that the repeated exposure to Nov, but not Soc, can counteract the Aβ_1-42_ effects without increasing the length of TH + axons.

Additionally, to determine the specific toxic effects of Aβ on TH + axons in the hippocampus CA1, we compared the effects of a random sequence of peptides of Aβ (SCR) and Aβ_1-42_ by intracranial infusion in a separate group of mice, called SH + SCR, n = 4; and SH + Aβ, n = 5. The SCR peptide comprises the same amino acids as Aβ but is arranged in a scrambled sequence to determine that it does not cause a toxic effect. Our stereological analysis showed that Aβ_1-42_, but not SCR peptides, induces the loss of TH + axons in the hippocampus CA1 (t-student test, SH + SCR vs. SH + Aβ; p < 0.01; Figure S1 A-B). Together, these observations suggest that the primary structure of Aβ_1-42_ is a crucial factor responsible for triggering the toxic effects.

## Novelty exposure maintains tonic dopamine levels within the hippocampus after Aβ_1-42_ oligomer administration

Previous evidence has shown that the overexpression of Aβ in an AD animal model impairs the catecholamine system (Moreno-Castilla et al. 2016; Nobili et al. 2017; La Barbera et al. 2021). Thus, we assessed whether acute Aβ_1-42_ administration to the treated groups affected catecholamine levels. We conducted in vivo microdialysis to evaluate levels of extracellular DA and NA, following infusion of either SS or Aβ in independent groups of mice, denominated SH + SS as a control group, n = 11; SH + Aβ, n = 4; Nov + SS, n = 7; and Nov + Aβ, n = 7, in the hippocampus CA1 (Fig. 4A). Based on our behavioral analysis and stereological quantification, we hypothesized that the Nov condition would improve catecholaminergic levels in the hippocampus CA1.

Consistently, the levels of DA in the Nov + SS group were higher than those in the SH + SS control group (one-way ANOVA, F (3, 25) = 9.378, p < 0.0001; post hoc test showed differences to SH + SS vs. Nov + SS, p < 0.0001, Fig. 4B). Similarly, we observed that the basal extracellular levels of DA in Nov + Aβ were higher than the SH + Aβ (SH + Aβ vs. Nov + SS, p < 0.0001, Fig. 4B). Interestingly, after Aβ_1-42_ oligomer infusion into the dorsal hippocampus in Nov-subjected mice, we observed similar extracellular levels of DA to the Nov + SS (Nov + SS vs. Nov + Aβ, p = NS, Fig. 4B). Notably, these basal DA levels are increased in Nov + Aβ mice compared to the SH + SS control group (SH + SS vs. Nov + Aβ, p < 0.05, Fig. 4B). In contrast, the extracellular DA levels did not change after Aβ infusion into the hippocampus between SH + SS and SH + Aβ (SH + SS vs. SH + Aβ, p = NS). This data suggests that the Nov protocol can increase DA extracellular levels, even after Aβ intrahippocampal infusion.

Next, we measured the basal extracellular levels of NA in the hippocampus CA1. We only found an increase in the Nov + SS condition compared with the SH + SS group (one-way ANOVA, F (3, 25) = 14.892, p < 0.0001; the post hoc test showed statistical differences between SH + SS vs. Nov + SS, p < 0.0001; Fig. 4C). Similarly, mice subjected to SH + Aβ showed lower extracellular levels of NA than Nov + SS mice (SH + Aβ vs. Nov + SS, p < 0.001). Next, we measured the NA extracellular levels in the Nov group. Our analysis showed significantly lower NA basal levels in Nov + Aβ compared to the Nov + SS group (Nov + SS vs. Nov + Aβ, p < 0.01, Fig. 4C). These results suggest that an increase in DA levels, but not NA levels, may be linked to preventing the Aβ_1-42_ toxic effects. It remains to be determined how these catecholaminergic differences are involved in counteracting the Aβ effects.

Finally, several works have found that the intrinsic excitability recordings within the hippocampus are modified after exogenous Aβ infusion (Tamagnini et al. 2015; Minkeviciene et al. 2009). Therefore, to explore possible changes in hippocampal neurochemical activity after Aβ infusion, we analyzed the extracellular glutamate (Glu) and γ-aminobutyric acid (GABA) levels in the same conditions described above. Interestingly, we did not detect changes in extracellular levels of Glu and GABA after any Soc or Nov protocol with or without Aβ_1-42_ infusion (one-way ANOVA, F (3, 25) = 2.13, p = NS, for Glutamate, Fig. 4D; and F (3, 25) = 1.055, p = NS, for GABA, Fig. 4E). Altogether, these results suggest that Nov condition specifically improves the extracellular levels of catecholamines, particularly the DA within the hippocampus CA1, preventing the Aβ toxic effects.

## Discussion

Clinical and experimental evidence has shown that the accumulation of Aβ in several brain areas can impair the activity of neurotransmitter systems and cognitive functions (Moreno-Castilla et al. 2016; Nobili et al. 2017; Takahashi et al. 2021; Wang et al. 2024; Gloria et al. 2021; Tolar et al. 2021). However, exposure to enriched environments has been proposed as a therapeutic tool to delay AD animal models' cognitive dysfunctions caused by Aβ load. In this regard, it has been shown that social environment or physical activity alone does not contribute to the behavioral benefits of enrichment environments that prevent cognitive dysfunction due to Aβ accumulation (Arendash et al. 2004). Therefore, it has been suggested that the novelty-driven environment may facilitate resilience development against the presence of Aβ. Nevertheless, it is unclear how repeated exposure to Nov can prevent Aβ damage in the CA1 region of the hippocampus.

## The novelty environment prevents the cognitive damage induced by Aβ_1-42_ oligomers

To determine whether the Nov could support the cognitive reserve hypothesis, we evaluated whether repeated exposure to Nov would prevent the effects of acute Aβ_1-42_ oligomer infusion in the hippocampus CA1 on spatial memory retrieval. After exposure to SH, Nov, and Soc conditions, all groups received SS or Aβ_1-42_ oligomer infusions within the hippocampus 24 h before the spatial memory test.

Our results starkly contrast the effects of Aβ_1-42_ oligomer administration in the SH + Aβ group and the Nov + Aβ group. MWM retrieval was impaired in the former, affecting the exact platform and quadrant positions compared to SH + SS control groups. However, the repeated exposure to a Nov environment demonstrated remarkable resilience against administering Aβ_1-42_ oligomers in the latter. The Nov + Aβ group performance was similar to the SH + SS control groups in the MWM test. This cognitive protection effect could be due to the Nov exposure but not by the Soc condition, which is associated with increased dopaminergic activity.

Consistent with our idea, it has been demonstrated that increased dopaminergic neurotransmission in the dorsal hippocampus is beneficial and necessary to improve spatial memory (Gálvez-Márquez et al. 2022; McNamara et al. 2014; Tse et al. 2023). Particularly, some studies have shown that a Nov significantly increased the release of both DA and NA necessary to encode contextual information in the hippocampus (Li et al. 2003; Moreno-Castilla et al. 2017; Gálvez-Márquez et al. 2022; Moreno-Castilla et al. 2018), and it was relevant to promote the persistence of a spatial memory task (Tse et al. 2023; Takeuchi et al. 2016). This evidence, as spatial memory is maintained in our experiments, suggests that enhanced DA activity is critical in preventing cognitive damage against Aβ_1-42_ toxic effects within the hippocampus CA1.

Several works have demonstrated, in AD patients and animal models, that noradrenergic and dopaminergic neurotransmission pathways innervating the hippocampus and the cortical brain areas are extensively affected by AD progression (Moreno-Castilla et al. 2016; Martorana and Koch 2014; Nobili et al. 2017; Herregodts et al. 1989; Torack and Morris 1992; Trillo et al. 2013). Our observations, in line with these findings, showed that the length of the TH + fibers in the CA1 region of the hippocampus decreases after injections of Aβ_1-42_. A similar study has demonstrated that the dosing of Aβ_1-42_ induced a significant shortening of the TH + fibers in the cortex without showing alterations after randomly administering similar peptides (Moreno-Castilla et al. 2016). In contrast, our stereological results suggest that repeated exposure to Nov may stimulate the release of catecholamines t preserve cognitive function against the hippocampal dosing of Aβ_1-42_. We suggest administering soluble oligomer forms Aβ_1-42_, even without amyloid plaques, can decrease the TH + axonal function, resulting in spatial recognition memory loss. Our observations are consistent with previous observations that show the Aβ oligomer can impair synaptic plasticity within the hippocampus (Tomiyama et al. 2010). Although it has been found that hyperphosphorylated Tau protein also impairs synaptic plasticity (Ondrejcak et al. 2018), the Aβ_1-42_ oligomeric forms are essential in AD progress due to precedes to pTau and intracellular neurofibrillary tangles (Roda et al. 2022). Therefore, we centered on Aβ_1-42_ oligomeric toxic effects and how the constant Nov exposure can represent an environmental factor maintaining cognitive performance against Aβ_1-42_ damage. Further experiments could help to understand the effects of Nov or Soc conditions against the pTau accumulation.

## Repeated novelty exposure enhanced dopaminergic activity enabling resilience to Aβ_1-42_ toxic effects

The evidence has demonstrated that environmental enrichment may protect against AD progression in animal models (Costa et al. 2007; Jankowsky et al. 2005; Arendash et al. 2004; Segovia et al. 2010; Naka et al. 2002). However, a cellular mechanism of brain protection against degeneration and cognitive dysfunction has yet to be identified. Recent studies have shown that animals exposed to enriched environments prevent deterioration of object recognition memory formation when Aβ oligomers are directly administered into the dorsal hippocampus. This prevention is associated with antioxidative stress and the expression of neurotrophic factors (Prado Lima et al. 2018). These observations have supported the hypothesis that repeated positive experiences are factors underlying enriched environments that positively affect the brain and may be related to cognitive resilience. Therefore, it is essential to understand these factors, as they may lead to interventions to build cognitive resilience and preserve cognition and memory in AD (Segovia et al. 2010).

A recent meta-analysis of AD patients showed significantly lower DA and NA levels (Pan et al. 2020; Dahl et al. 2023), similar to a transgenic AD mouse model in diverse cortical brain areas and the hippocampus (Moreno-Castilla et al. 2016; Nobili et al. 2017). Interestingly, after exposing wild-type animals to enrichment environment protocols for several weeks, the biochemical analysis revealed an increased release of catecholamines into the cortex, the cerebellum, and the nucleus accumbens (Segovia et al. 2010; Naka et al. 2002). Therefore, to identify a possible role of the higher catecholamine levels in the hippocampus as one of the elements involved in the cognitive reserve, we performed microdialysis in freely moving mice to assess whether a repeated Nov may increase the catecholamine levels. Consistently, our Nov protocol significantly increases the basal concentration of DA and NA in the dorsal hippocampus. Notably, the Aβ oligomers infusion did not affect the DA levels in the Nov + Aβ group compared to the SH + SS control mice. Unlike increased DA levels, the Glu and GABA extracellular concentrations remained similar across all groups. According to previous reports, these results support the idea that repeated Nov exposure may specifically increase catecholamine levels. Thus, the catecholaminergic system, particularly the DA activity, could mediate the resilience against Aβ oligomers and allow us to propose that repeated Nov exposure may create cognitive resilience.

Several works have found that the activity of catecholaminergic fibers in the dorsal hippocampus is necessary to enable spatial memory (Gálvez-Márquez et al. 2022; McNamara et al. 2014; Tse et al. 2023). Thus, the increase in the release of both DA and NA within the hippocampus through TH + axonal photostimulation fibers of the locus coeruleus can modulate spatial memory retrieval (Gálvez-Márquez et al. 2022; Tse et al. 2023). Moreover, it has been found that the DA agonist also improves spatial memory performance in AD transgenic models (Himeno et al. 2011; Ambrée et al. 2009). Consistently, the enriched environment exposition restores the positive immune signal to TH + in the substantia nigra neuronal bodies in a model of Parkinson’s disease (Goldberg et al. 2011). However, although the TH + axonal projections into the cortical brain areas or the DA release into the nucleus accumbens is decreased in the presence of Aβ (Moreno-Castilla et al. 2016; Nobili et al. 2017), it is unclear whether the Aβ can impair the TH + axonal projections integrity or specifically the catecholamine synthesis. In this regard, it has been observed that the Aβ could alter the phosphokinase C activity, decreasing the transcriptional activity factors such as cAMP response element-binding (CREB) (Zhong et al. 2003). This factor can regulate the mRNA synthesis of several proteins involved in neurotransmission, such as the TH enzyme, and trophic factors involved in neuroplasticity and neuronal survival, like the brain-derived neurotrophic factor (BDNF) (Tank et al. 2008). In consistency, it is known that the expression of neurotrophic factors decreases in AD patients (Zuccato and Cattaneo 2009), which in experimental models is enhanced after exposure to an enriched environment (Jha et al. 2011) or intrahippocampal injections of rotigotine, a DA receptor agonist (Adachi et al. 2018). A recent report found that DA and NA can prevent Aβ oligomerization in vitro conditions (Allnutt and Matera 2024). Therefore, these observations suggest that the Nov protocol can increase the basal DA and NA levels, even in the presence of Aβ oligomers. Suppose DA and NA can prevent the oligomerization in in vitro conditions. In that case, it is likely to impede the intrahippocampal Aβ oligomeric stabilization, decreasing their toxicity effects and favoring the inherent neuronal plasticity, for example, through BDNF expression. Therefore, we proposed that establishing a resilient effect against Aβ oligomers could be mediated by direct catecholaminergic activity interaction with Aβ oligomers within the hippocampus. However, the specific mechanisms by which DA can protect against the toxic Aβ oligomers are a subject for further research.

In contrast, the Soc conditions showed significantly lower performance in the MWM after Aβ was administered and non-recovery of + TH fibers and extracellular levels of DA within the dorsal hippocampus. Although previous works have found that the Soc conditions can elicit dopaminergic activity (Kopec et al. 2019), decreasing the harmful cognitive effects in AD patients (Bennett et al. 2006; Fratiglioni et al. 2000), our data has not shown a protective factor against the Aβ. These observations could be due to the inherent conditions of our Soc protocol, which is that the Nov factor is not included. Across the intra- and intersessions of Soc training, all animals are habituated to the same fellow mice, sidelining the Nov factor and decreasing the DA activity (Tops et al. 2013; Leussis and Bolivar 2006). In contrast, the novelty factor is present in the Nov group because the objects and their positions constantly changed between intersessions, allowing increased DA activity.

Additionally, these observations are consistent with the length of the TH + fibers diminished in Soc mice, but not in the Nov group, after Aβ oligomers administration, supporting the idea that the catecholaminergic activity, particularly the DA system, can improve the spatial memory recall after dosing Aβ in the hippocampus. Our result discloses that the Nov, but not the Soc conditions, may play a key role in the resilience to Aβ toxic effects. These observations contribute to explaining how the Nov may be a key element in forming the cognitive reserve hypothesis and opening new therapeutic tools to prevent and delay AD signs, especially in the early stages of this neurodegenerative disease.

## Conclusions

Our research underscores the significant impact of Aβ oligomers on the catecholaminergic system, as evidenced by the reduction in TH + fiber length and baseline levels, affecting spatial memory task performance. However, we also demonstrate that these cognitive impairments can be reversed by stimulating catecholaminergic activity through exposure to a Nov. This suggests that lifestyle choices and experiences, such as exposure to Nov, could serve as a natural and effective treatment for preventing AD outcomes by stimulating the dopaminergic system.

## Supplementary Information

Below is the link to the electronic supplementary material.

Supplementary file1 (2.89 MB)

## Abbreviations

AD: Alzheimer´s Disease
SH: Standard housing
Nov: Novelty stimuli
Soc: Social interaction
DA: Dopamine
NA: Noradrenaline
Aβ: Beta-amyloid peptide
TH: Tyrosine hydroxylase
