# Supplementary material for: Repeated exposure to novelty promotes resilience against the amyloid-beta effect through dopaminergic stimulation
Source: Psychopharmacology (Berl). 2024 Aug 15;242(1):85–100. doi: 10.1007/s00213-024-06650-5 (PMC11742894; doi:10.1007/s00213-024-06650-5)
Supplement: Supplementary file 1 — Supplementary file1 (2.89 MB) [file 213_2024_6650_MOESM1_ESM.docx]

**Supplemental information**

**Figure S1**


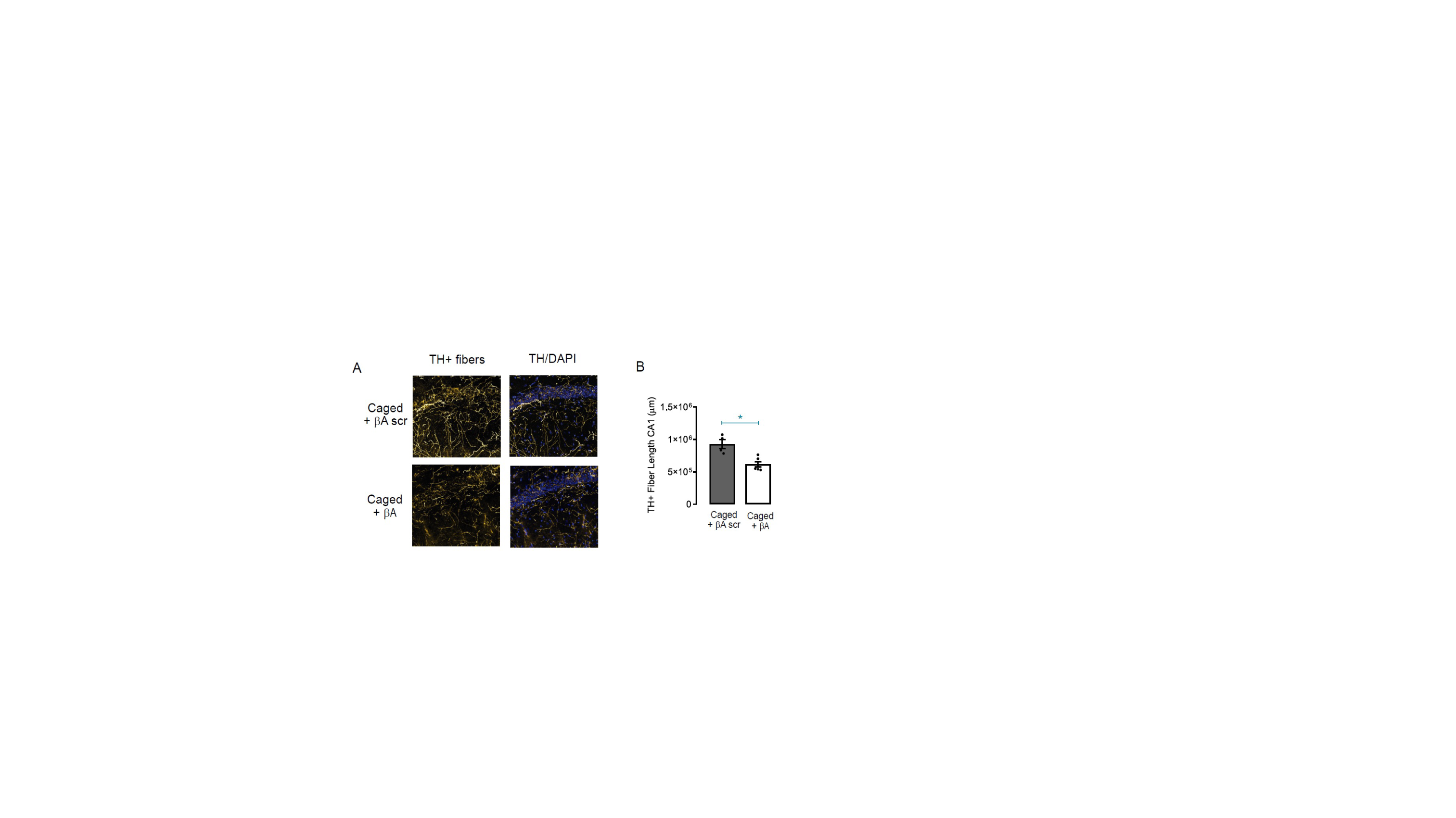

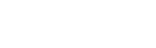

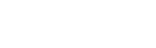

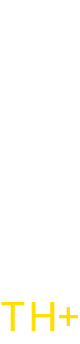

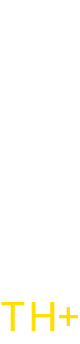

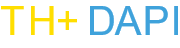

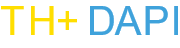

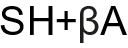

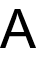

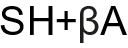

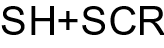


TH+ Fiber length CA1 (µm)


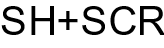


**Figure S1. The SCR sequence of Aβ did not affect the TH+ length within the dorsal hippocampus.** A) Depicts the confocal photomicrography of TH+ axons (yellow) and nuclei of the dorsal hippocampus after SH + SCR (top-row) and SS + Aβ_1-42_ groups (bottom-row). B) Show the TH+ fiber length within the hippocampus-CA1 measured by stereological quantification. Data are shown in ±SEM, ***p < 0.05.**

**Figure S2**


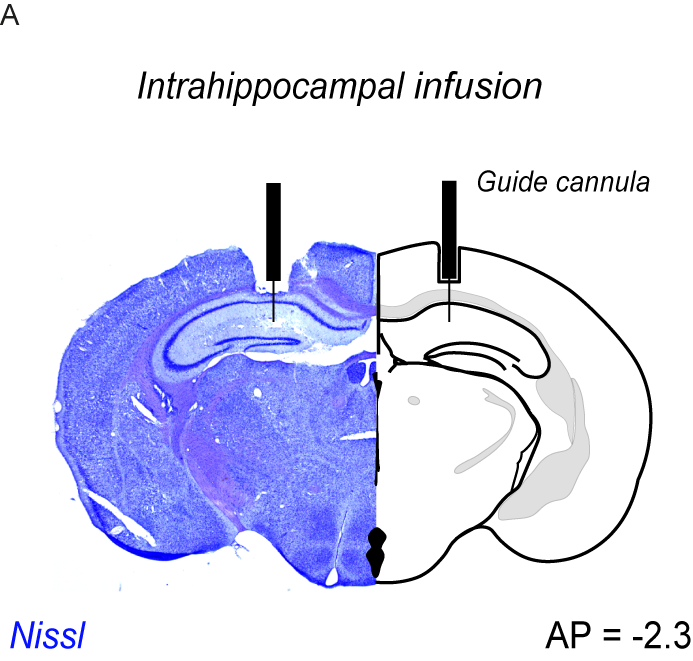


**Figure S2. Bilateral intrahippocampal administration.**A) Representative micrograph of the bilateral cannula implantation site in the dorsal hippocampus (AP from Bregma -2.3). The cellular bodies were processed with Nissl staining (purple). All animals receiving an intrahippocampal infusion of saline or Aβ were implanted using the described coordinates in the Methods section.
